# Species Delimitation of Asteropyrum (Ranunculaceae) Based on Morphological, Molecular, and Ecological Variation

**DOI:** 10.3389/fpls.2021.681864

**Published:** 2021-09-10

**Authors:** Shanmei Cheng, Weidong Zeng, Jing Wang, Lei Liu, Hua Liang, Yixuan Kou, Hengchang Wang, Dengmei Fan, Zhiyong Zhang

**Affiliations:** ^1^Laboratory of Subtropical Biodiversity, Jiangxi Agricultural University, Nanchang, China; ^2^CAS Key Laboratory of Plant Germplasm Enhancement and Specialty Agriculture, Wuhan Botanical Garden, Chinese Academy of Sciences, Wuhan, China; ^3^Center of Conservation Biology, Core Botanical Gardens, Chinese Academy of Sciences, Wuhan, China

**Keywords:** leaf shape, leaf size, geometric morphometrics, Automatic Barcode Gap Discovery, niche modeling, species delimitation, integrative taxonomy, Asteropyrum Drumm. et Hutch.

## Abstract

Objectively evaluating different lines of evidence within a formalized framework is the most efficient and theoretically grounded approach for defining robust species hypotheses. Asteropyrum Drumm. et Hutch. is a small genus of perennial herb containing two species, A. cavaleriei and A. peltatum. The distinction of these two species mainly lies in the shape and size of leaf blades. However, these characters have been considered labile and could not differentiate the two species reliably. In this study, we investigated the variation of the leaf blades of 28 populations across the whole range of Asteropyrum using the landmark-based geometric morphometrics (GMM), sought genetic gaps within this genus using DNA barcoding, phylogenetic reconstruction and population genetic methods, and compared the predicted ecological niches of the two species. The results showed that the leaf form (shape and size) was overlapped between the two species; barcode gap was not detected within the genus Asteropyrum; and little ecological and geographical differentiation was found between the two taxa. Two genetic clusters detected by population genetic analysis did not match the two morphospecies. The results suggest that there are no distinct boundaries between the two species of Asteropyrum in terms of morphology, genetics and ecology and this present classification should be abandoned. We anticipate that range-wide population genomic studies would properly delineate the species boundaries and help to understand the evolution and speciation within Asteropyrum.

## Introduction

Delimiting species boundaries correctly is crucial to the discovery of life’s diversity because it determines whether or not different individual organisms are members of the same entity (Dayrat, 2005). Traditionally, species are mostly established based on morphology which Cain (1954) called “morphospecies.” However, under the unified species concept that defines species as segments of population-level evolutionary lineages (De Queiroz, 2007), distinctive morphology, reciprocal monophyly for haplotypes, reproductive isolation, and divergent ecology can arise at different times and in different orders during the process of speciation (De Queiroz, 1998; Leaché et al., 2009). Thus morphospecies can only be seen as hypotheses that should be tested via different approaches and by using multiple lines of evidence (De Queiroz, 2007). Furthermore, some taxonomists describe species typologically that ignores intraspecific variation and morphological plasticity, this practice could lead to oversplitting by misinterpreting individual variants as members of new specific entities (Dayrat, 2005). The oversplit morphospecies may result in an overestimation of biodiversity and then a waste of conservation resources (Hong, 2016). Therefore, morphospecies with great intraspecific variation and morphological plasticity are particularly in need of reevaluation with respect to their morphology, genetics, and ecology.

Leaf morphology is very important to plant taxonomy and systematics and the variation of leaf form has mostly been studied using morphometrics (Jensen, 2003). Traditional morphometrics involves the application of multivariate statistical analyses to collections of distances, angles, or distance ratios (Adams et al., 2004), seldom considering the shape information (Rohlf and Marcus, 1993). Landmark-based geometric morphometrics (GMM), however, provides a viable alternative for analyzing complex shapes in multivariate space by retaining information about the relative spatial arrangements of the landmarks, allowing for the visualization of shape differences among groups (Zelditch et al., 2004). During the last decade, leaf form variability has fruitfully been investigated using GMM to accurately discriminate species and their hybrids (Viscosi and Cardini, 2011).

DNA sequence-based methods for species delimitation have become popular in recent years. DNA barcoding, an approach initially being developed to identify species based on sequences from a short, standardized DNA region (Hebert et al., 2003; Hebert and Gregory, 2005), are proving to be a robust tool to help unveil biodiversity in a fast, accurate way relying on a distance threshold or “barcoding gap” (e.g., Rossini et al., 2016). However, species delimitation based on a single barcode may be an inaccurate portrait of speciation history (Maddison, 1997). DNA barcoding approaches of species delimitation are often complemented with population genetic analyses, especially those based on multiple nuclear loci (e.g., Lu et al., 2021).

Ecological niche modeling (ENM) utilizes associations between environmental variables and known species’ occurrence localities to define abiotic conditions within which populations can be maintained (Guisan and Thuiller, 2005). ENM has already been integrated into a broad variety of research disciplines including species delimitation (Sites and Marshall, 2003; Raxworthy et al., 2007). If a set of populations is geographically separated from closely related species with similar ecological niche by unsuitable habitats, this pattern would support the hypothesis of allopatric speciation with niche conservatism (Peterson et al., 1999). Alternatively, if a set of populations occurs under climatic conditions that are different from closely related species, then gene flow between these populations and other species may also be unlikely or restricted, and these populations may represent a distinct species (Wiens and Graham, 2005).

**FIGURE 1:**
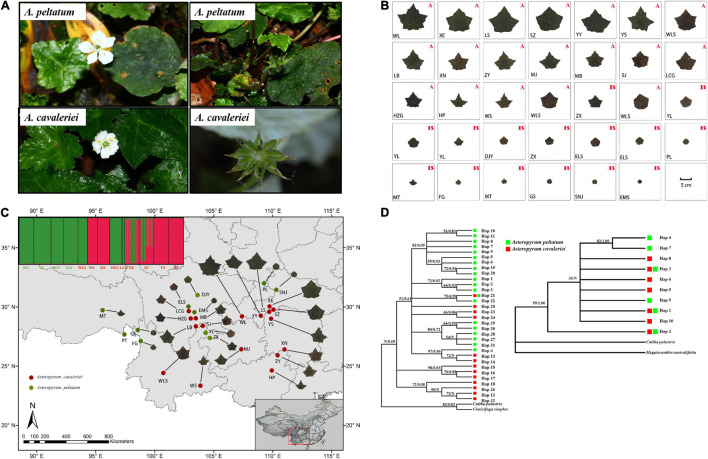
The morphological characters of flowers and fruits **(A)** and the continuous morphological variation of leaf form in 27 populations of Asteropyrum **(B)** and their geographic distribution **(C)**, the capital letters A and B in **(B)** and the populations marked in red and green font in **(C)** represent A. cavaleriei and A. peltatum, respectively. Maximum likelihood and Bayesian consensus trees of nrITS2 ribotypes (left) and psbA-trnH chlorotypes (right) of Asteropyrum [**(D)**, ML bootstrap values above 50% and posterior probability are shown on the branches, N represent low supporting values].

Asteropyrum J. R. Drummond et Hutchinson is a small genus of perennial herb in the family Ranunculaceae, comprising two species, A. cavaleriei (Lévl. et Vant.) Drumm. et Hutch. and A. peltatum (Franch.) Drumm. et Hutch. (Fu and Robinson, 2001). Originally, the two species were described as members of Isopyrum L., however, they have simple leaves, differing obviously from the bi-ternately compound leaves of Isopyrum species. Thus, Asteropyrum was later established by Drummond and Hutchinson (1920) to accommodate the species with simple leaves. The leaf blade of A. cavaleriei is deeply five-lobate, with a width of 4–14 cm, but that of A. peltatum is inconspicuously lobate and 1–3.7 cm wide. In addition, the scape of the former (12–20 cm) is much longer than that of the later (6–10 cm) (thereafter the two species are referred to as “morphospecies”). However, with regard to their gross morphology and karyotype, A. peltatum and A. cavaleriei are very similar (Figure 1A and Supplementary Figure 1), some populations are intermediate in leaf shape and size (Yuan and Yang, 2006). Accordingly, Yuan and Yang (2006) treated the two morphospecies as subspecies of A. peltatum. Although Yuan and Yang (2006) recognized that the morphological differentiation between A. peltatum and A. cavaleriei is obscure, they had not conducted statistical analyses on the leaf morphology and had not evaluated their genetic and ecological differentiation. In our pilot investigations, we even found that the two morphospecies occur in the same populations occasionally. These field observations cast new doubt on the taxonomic status of the two morphospecies or even morpho-subspecies. Therefore, the boundary between the two Asteropyrum species should be tested by different lines of evidence, including GMM, DNA barcoding, population genetics, and ecological niche modeling.

In this study, we extended our investigation to 28 wild populations, then conducted a geometric morphometric analysis on leaf form and quantified genetic and ecological niche divergence of the two morphospecies. Our specific objectives are: (1) to investigate the morphological variation of Asteropyrum across its distribution range; (2) to test if the two morphospecies of Asteropyrum are independent by means of GMM, DNA barcoding, population genetics, and ecological niche modeling.

## Materials and Methods

### Field Investigation and Sample Collection

We searched for distribution information of Asteropyrum from Chinese Virtual Herbarium (CVH) and websites of nature reserves or national parks. Most recorded locations have been investigated during the last 3 years. In total, fresh leaves of 28 populations were immediately dried with silica gel (Supplementary Table 1). Sampled individuals were spaced by >10 m to avoid sampling the ramets of the same genet. Geographical information (latitude, longitude, and altitude) was recorded with a smart phone’s GPS.

Four to six specimens in each population, and three mature leaves per specimen were sampled for morphological analyses. These mature leaves, each is the largest one from a randomly selected individual, were selected for avoiding developmental plasticity. Because the distinction between the two morphospecies is obscure, species identity was predefined with blind test by three experienced taxonomists according to the majority rule. All voucher specimens were stored at Jiangxi Agriculture University (JXAU).

### Morphological Analysis

**FIGURE 2:**
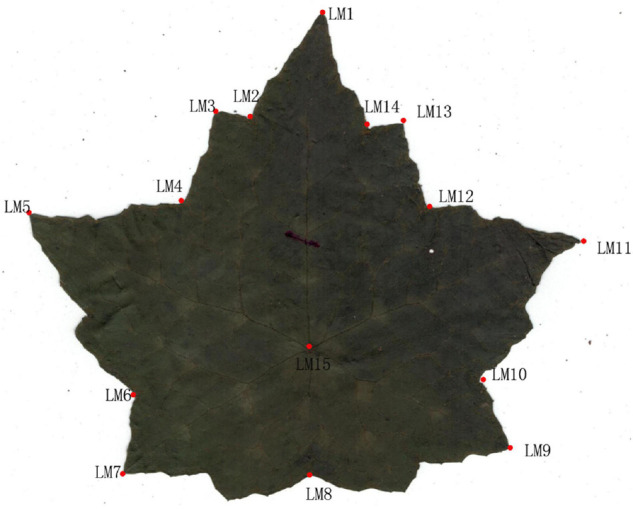
Fifteen landmarks on Asteropyrum leaves.

A total of 437 dry leaves from 27 populations were collected for morphological studies. The PT population was not measured owing to lack of samples and specimen. Leaves were pressed, dried and scanned with the adaxial uppermost using a scanner (Brother DCP-B7530DN) with a resolution of 300 dpi. Scanned images were used to record 15 landmarks (Figure 2 and Table 1 for detailed landmark definitions) following Viscosi and Cardini (2011). Cartesian coordinate data of 15 landmarks were acquired using software from the TPS Series (Rohlf, 2010) and then imported into the free software MorphoJ v. 1.04a (Klingenberg, 2011). For the landmark data, a generalized Procrustes analysis (GPA) was performed to extract shape and size components of form variation (Viscosi and Cardini, 2011), calculating the Procrustes distance (Rohlf and Slice, 1990) and Centroid distance (Loy et al., 1998), respectively. Procrustes distance and centroid distance are used to test the shape variation (Holling, 1992; Mitteroecker and Gunz, 2009) and size variation (Loy et al., 1998) between groups, respectively.

**TABLE 1:** Description of the landmarks of Asteropyrum leaves.

**Landmakers**	Description
LM1	Blade tip serrated point
LM2	The first deep depression on the left side of the blade from the tip
LM3	The first serrated point on the left side of the leaf from the tip
LM4	A second deep depression from the tip of the leaf
LM5	Highest serrated point on the left side of blade
LM6	Blade left highest sawtooth point down the deepest sag point
LM7	Blade left highest serrated point down the deepest sag point next serrated point
LM8	The lowest concave point of blade
LM9	A zigzag point symmetric with LM7
LM10	A concave point symmetrical to LM6
LM11	A zigzag point symmetric with LM5
LM12	A concave point symmetrical to LM4
LM13	A zigzag point symmetric with LM3
LM14	A concave point symmetrical to LM2
LM15	The central point of the vein between leaf blade and stem

For Procrustes distance, Kolmogorov–Smirnov normal test (K-S test), MANOVA test (multivariate analysis of variance) at intraspecies and interspecies levels were carried out (Hair et al., 1998; Limsopatham et al., 2018; Hošková et al., 2021). For centroid distance, K-S normal test, ANOVA test (univariate analysis of variance) were carried at the same levels as well. SPSS 22.0 software was used for these statistical analyses.

Using the variance-covariance matrix of the GPA shape coordinates, we carried out a principal component analysis (PCA), a canonical variate analysis (CVA) and a discriminant analysis (DA) for leaf shape. All of the analyses were accomplished in software MorphoJ v. 1.04a (Klingenberg, 2011).

### Molecular Procedure and Species Delimitation Based on DNA Sequences

Total genomic DNA was extracted from silica-dried leaves using a modified cetyltrimethylammonium bromide (CTAB) method (Doyle and Doyle, 1987). We amplified and sequenced DNA barcoding markers (intergenic spacer psbA-trnH and internal transcribed spacer 2, ITS2) across all sampled Asteropyrum populations. To complement the barcoding analyses, five low-copy nuclear loci were also amplified to evaluate the genetic divergence at nuclear genome, with A. cavaleriei and A. peltatum represented by nine and four populations, respectively. The primers used for the polymerase chain reaction (PCR) followed Sang et al. (1997) for psbA- trnH and White et al. (1990) for ITS2, and the primers of five nuclear loci were designed from transcriptome sequences following our previous study (Kou et al., 2020). Amplification reactions were carried out in a volume of 20 μl, containing 10 μl 2 × Taq PCR MasterMix (Tiangen, Shanghai, China), 1 μl each forward and reverse primer (0.2 μM), 1 μl template DNA (ca. 50–100 ng) and 7 μl ddH_2_O. Amplification was carried out in a Bioer XP cycler (Bioer, Hangzhou, China) programmed for an initial step 5 min at 94°C, followed by 36 cycles of 94°C for 50 s, 50–53°C for 50 s and 72°C for 1 min or 1 min and 40 s, with a final extension for 10 min at 72°C. Sanger sequencing reactions were conducted with the corresponding forward and reverse primers commercially by Sangon Biotech Co., Ltd. (Shanghai, China).

All sequences were edited with Sequencher (GeneCodes Corporation, Ann Arbor, MI, United States), aligned using BioEdit 7.2 (Hall, 1999) and refined manually in MEGA v. 5.05 (Tamura et al., 2011). Genetic diversity and neutrality tests were estimated in DnaSP v5.10 (Librado and Rozas, 2009). According to two recent phylogenetic studies (He et al., 2019; Zhai et al., 2019), Caltha L. or Caltha-Delphinieae-Nigelleae may be the closest relative of Asteropyrum, so we selected Caltha plus a more distant genus (Cimicifuga for psbA-trnH phylogeny and Megaleranthis for ITS2 phylogeny) as outgroups. Those corresponding sequences of outgroups were downloaded from GenBank (accession numbers: KP643431, FJ597983, KX167189, and GQ351363).

The best substitution model was respectively, selected using jModeltest 2 (Darriba et al., 2012) for psbA-trnH and ITS2 haplotype matrices and phylogenetic inferences were carried out using PhyML v3.0 (Guindon et al., 2010) with 1,000 bootstrap pseudo replicates for estimation of the branch support. Bayesian phylogenetic analysis using MrBayes 3.2 (Ronquist et al., 2012) were also performed (mcmcp ngen = 1000000, burnin = 250). The resulting maximum likelihood (ML) and Bayesian trees were visualized with FigTree v1.4.4 (Rambaut, 2018). To define species on the basis of psbA-trnH and ITS2 haplotypes, the Automatic Barcode Gap Discovery (ABGD) species delimitation tool was employed (Puillandre et al., 2012). Briefly, ABGD is an automatic procedure that sorts the sequences into hypothetical species based on the barcode gap. It uses a range of prior intraspecific divergence to infer from the data a model-based one-sided confidence limit for intraspecific divergence. The method then detects the barcode gap as the first significant gap beyond this limit and uses it to partition the data. Inference of the limit and gap detection are then recursively applied to previously obtained groups to get finer partitions until there is no further partitioning (Puillandre et al., 2012). We used MEGA v5.05 (Tamura et al., 2011) to compute pairwise sequence comparison matrices using the p-distance, Kimura-2-parameter (K2P) corrected distance (D) (Kimura, 1980); the matrices were then analyzed in the ABGD online species delineation tool^1^.

Pairwise differences between different populations and haplotypes were calculated by software Arlequin v3.0 (Excoffier et al., 2005). These were used for mantel test with GenALEX v6.5 (Peakall and Smouse, 2012) and to construct a miniman-spanning network by Network 10.3^2^, respectively. Structure 2.3.4 (Hubisz et al., 2009) was used to assess population structure with the admixture model and the assumption of correlated allele frequencies using the dataset of five low-copy nuclear loci. The number of clusters, K, corresponding to the number of populations, was explored using 20 independent runs per K. Burn-in was set to 20000 and Markov chain Monte Carlo (MCMC) run length to 200000. We ran Structure with K varying from 1 to 10, with 10 runs for each K value. The most likely number of clusters was estimated using LnP (D) (Pritchard et al., 2000) and ΔK statistics (Evanno et al., 2005). The population clusters were visualized using the program Distruct 1.1 (Rosenberg, 2004).

### Ecological Niche Modeling

According to the geographical coordinate of the presence sites, the corresponding 19 bioclimatic variables were got through DIVA-GIS software Version 7.5.0^3^. After the standardized processing of the bioclimatic matrix data, a principal component analysis was carried out in SPSS 22.0 to test whether the morphospecies are discernible based on the climatic variable. The eigenvalue >1 was selected as the principal component extraction method. The “factor score” was saved as a variable and converted into “principal component score” for scatter plot analysis. Meanwhile, the correlation analyses were also performed among 19 bioclimatic variables and applied a correlation threshold of 0.8 to select variables for ecological niche modeling.

In order to examine niche divergence between the two morphospecies of Asteropyrum, we used Maxent v3.3.3k (Phillips et al., 2006) to generate predictive distribution models based on known occurrence records and their corresponding environmental variables (compiled from the WORLDCLIM database with a resolution of 2.5 arc-min) using Maxent (Elith et al., 2006). Niche modeling was constructed for present-day, the Last Glacial Maximum (LGM) and the Last Interglaciation (LIG) periods using 150 presence records that include the sampling sites of this studies and herbarium specimen records from Chinese Virtual Herbarium (CVH). The models were run 10 times using the parameters (convergence threshold of 10^–5^, maximum iterations of 5000 and regularization multiplier of 10) and the following user-selected features: application of a random seed, duplicate presence records removal and logistic probabilities used for the output. Eighty percent of the presence records being used for training and 20% for testing the model. The models were then projected onto a broader geographic area encompassing East Asia. The calculated AUC (the Area Under the Receiver Operating Characteristic Curve) values of the Receiver Operating Characteristics (ROC) curve were used to assess the accuracy of the simulation (Elith et al., 2006). A score above 0.9 indicates that the simulation model has high accuracy and reliable results (Fielding and Bell, 1997). Maps were generated using ArcGIS Version 9.3^4^. In addition, we estimated the degree of niche overlap between the two morphospecies with ENMTools Ver. 1.4.4 (Warren et al., 2010) based on Schoener’s D (Schoener, 1968).

## Results

### Geometric Morphometric Analysis

Kolmogorov–Smirnov normal test was carried out on Procrustes distance and centroid distance, respectively. The results showed that the distance data used in this study were normally distributed (P > 0.05). Multivariate analysis of variance (MANOVA) test on Procrustes distance showed very significant multivariate differences at both inter- and intraspecific levels (P < 0.001, Table 2). Differences between individuals (63.4% of explained sum of squares) were greater than between species (10.1%) and populations (26.5%, Table 2). For centroid distance, univariate analysis of variance (ANOVA) test showed that inter- and intraspecific differences were both significant as well, and difference between species (43.0%) were greater than between populations (24.6%) and individuals (32.4%, Table 2). These results are in contrast with the prediction that interspecific divergence would be larger than intraspecific divergence if there were morphological gaps between species.

**TABLE 2:** The results of multivariate analysis of variance (MANOVA) test on Procrustes distance and univariate analysis of variance (ANOVA) test on centroid size.

**Leaf character**	**Effect**	**SS**	**SS explained**	**MS**	**df**	**F**	**P**
Shape	Species	0.343306	10.1%	0.013204	26	27.40	<0.0001
	Population	0.899277	26.5%	0.001441	624	8.67	<0.0001
	Individual	2.155222	63.4%	0.000482	4472	2.19	<0.0001
Total		3.397805	100%				
Size	Species	39487527.50	43.0%	39487527.50	1	228.35	<0.0001
	Population	22599487.91	24.6%	941645.33	24	12.35	<0.0001
	Individual	29742946.76	32.4%	172924.11	172	3.68	<0.0001
Total		91829962.17	100%				

*SS, MS, df, F, and P represent sum of squares, mean sum of squares (i.e., SS divided by df), degrees of freedom, Goodall’s F statistic and p-values, respectively.*

**FIGURE 3:**
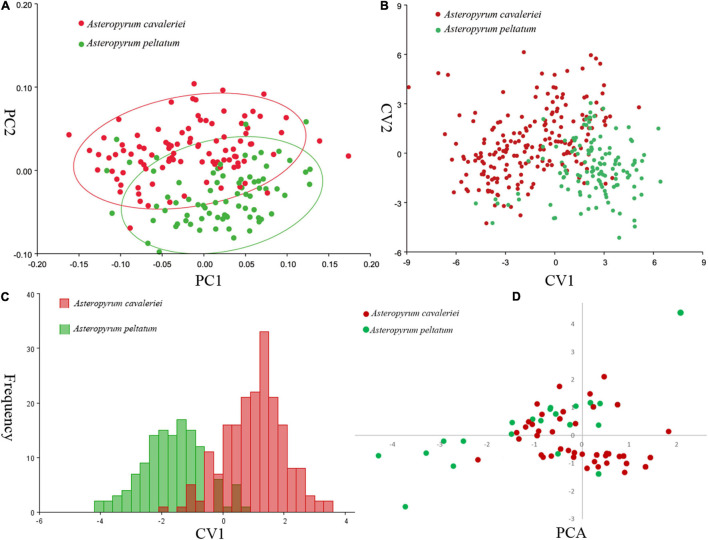
Comparisons of leaf shape of two morphospecies of *Asteropyrum* using principal component analysis (PCA, **A**), canonical variate analysis (CVA, **B**), discriminant analysis (DA, **C**), respectively. Multivariable plots of the 19 bioclimatic variables in *Asteropyrum* using PCA method **(D)**.

Principal component analysis (PCA) on the variance-covariance matrix of the Procrustes shape coordinates showed that the first three principal components explained 68.1% of the total variance. The scatter plots of PC1 vs. PC2 showed that the two morphospecies were partially overlapped (Figure 3A). Similarly, the first three canonical variables explained 69.8% of the total variance and the two morphospecies cannot be differentiated in CVA because their scatter plots were also partially overlapped (Figure 3B). As in the PCA and CVA, the results of the DA indicated that the range of leaf shapes (Figure 3C) and leaf size (data not shown) partially overlapped between the two morphospecies. The cross-validation classification showed that the accuracy of leaf shape in predicting species is better than a 86.99% random chance.

We arranged the picture of one representative leaf of each population together, the pictures visually display that the leaf of Asteropyrum varies continuously in shape and size (Figure 1B) and there is no morphological gap that are fit for delimiting species. We also generated a distribution map of leaf form (Figure 1C), the map shows that the leaf form of Asteropyrum has no obvious geographical pattern.

### Molecular Species Delimitation

We obtained sequences of the cpDNA intergenic spacer psbA-trnH from 313 individuals of 28 populations. The aligned sequence matrix was 219 bp in length, containing 15 substitutions. The substitutions defined 10 different haplotypes. ITS2 sequences were obtained from the same individuals and the aligned sequences were 260 bp in length. Forty-nine substitutions determined 32 haplotypes. All haplotype sequences are deposited in GenBank. Both haplotype diversity (H_d_ = 0.849, 0.909 for cpDNA and ITS, respectively) and nucleotide diversity (π = 0.011, 0.030 for cpDNA and ITS, respectively) are relatively high (Table 3). The neutrality tests including Tajima’s D and Fu and Li’s test showed that the genetic variabilities were not due to natural selection (Table 3).

**TABLE 3:** The genetic diversity and neutrality tests of six nuclear loci and one chloroplast fragment used in this study.

**Primers**	**Sequences (5′-3′)**	**L**	**S**	**π**	**N_h_**	**H_d_**	**D**	D*	F*
psbA-trnH	F:GTTATGCATGAACGTAATGCTC	219	15	0.011	10	0.849	0.491	1.453	1.309
	R:CGCGCATGGTGGATTCACAAATC								
ITS	F:GCTGCGTTCTTCATCGATGC	260	49	0.030	32	0.909	0.229	1.755*	1.257
	R:GGAAGTAAAAGTCGTAACAAGG								
X14	F:GTTTCGGGTGTTCTTGTT	388	16	0.005	19	0.827	−0.680	1.046	0.468
	R:CATCTTCTTGGCTCGTAG								
X35	F:CCGCTTTGCCACAGATTA	253	10	0.009	11	0.776	−0.200	1.344	1.114
	R:TGCTTTACCAGCCGTTGA								
X47	F:ACAACATCCCAATCAGCA	231	17	0.017	16	0.869	−0.039	−0.317	−0.251
	R:ACAACCCACAACACCAGA								
X63	F:CGTCGCCCAGTAGTATCTT	389	21	0.004	15	0.843	−1.580	1.304	0.211
	R:ACATTCATCGTTCGCTTG								
X130	F:GGGAAGCCGTAGACTCAC	275	17	0.006	16	0.790	−1.280	1.160	0.266
	R:CCCGACAAGGCATAGAAC								

L, length in base pair; S, number of polymorphic sites; π, nucleotide diversity; N_h_, number of haplotype; H_d_, haplotype diversity; D, Tajima’s D statistic; D* and F*, Fu and Li’s test statistic. The asterisk indicate statistical significances (P < 0.05).

**FIGURE 4:**
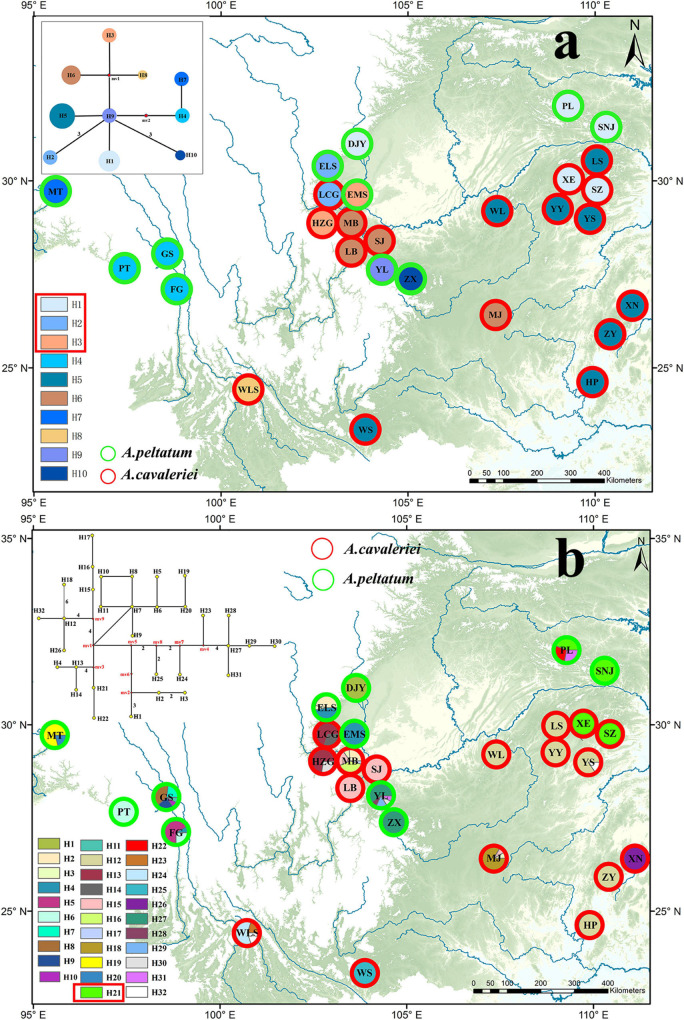
The distribution maps and network analyses of chlorotypes (psbA-trnH, **a**) and ribotypes (ITS, **b**). The haplotypes circled with red rectangle are shared by the two morphospecies.

Because psbA-trnH and ITS2 have a relatively rapid evolution rate in Ranunculaceae, it is difficult to align the psbA-trnH and ITS2 sequences between Asteropyrum and outgroups, especially when Cimicifuga (psbA-trnH phylogeny) and Megaleranthis (ITS2 phylogeny) were included. However, the topology of the haplotypes of Asteropyrum did not change irrespective of which outgroup(s) were used, thus all the outgroups were kept in further analyses without any exclusion of sequence regions. Maximum likelihood (ML) and Bayesian trees of psbA-trnH and ITS haplotypes indicated that the two Asteropyrum morphospecies were not reciprocally monophyletic, albeit most clades did not receive strong bootstrap support (Figure 1D). Likewise, the network analyses of psbA-trnH chlorotypes and ITS2 ribotypes also did not support the monophyly of the two morphospecies (Figure 4). Distributions of chlorotypes and ribotypes did not display obvious geographical or species-specific pattern (Figure 4).

The result of mantel test based on ITS2 sequences showed that the correlation coefficient was very low (r = 0.303, P < 0.01) between genetic distances and geographical distances. The mantel test of cpDNA data was not performed due to lack of informative sites.

Five low-copy nuclear loci were sequenced for 134 individuals from 13 populations. The total aligned length was 1,536 bp, with loci ranging from 231 to 389 bp. A total of 99 segregating sites, including two indels and 68 singleton sites after excluding the sites of significant linkage disequilibrium were used to population genetic structure analysis. The Bayesian clustering algorithm indicated that the most likely number of clusters was 2, however, the two genetic clusters did not correspond to the two morphospecies (Figure 1C).

**FIGURE 5:**
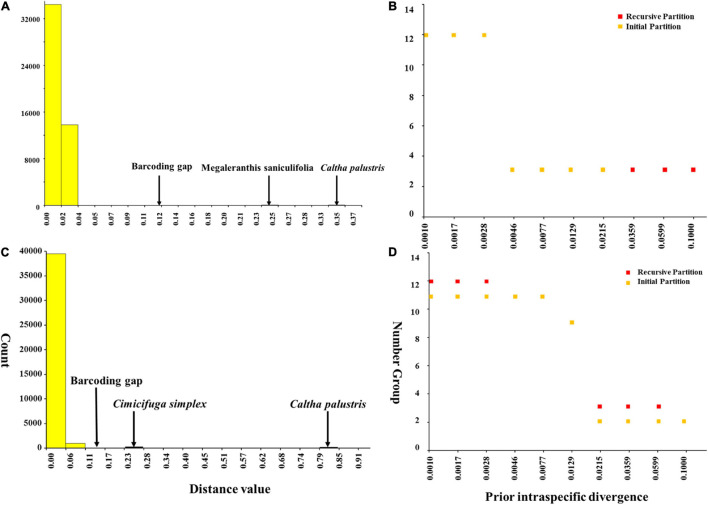
Barcoding gap and number of groups (species) inferred from Automatic Barcode Gap Discovery (ABGD). A hypothetical distribution of pairwise differences based on psbA-trnH **(A)** and nrITS sequences **(C)**, the number of species according to the prior intraspecific divergence based on psbA-trnH **(B)** and nrITS sequences **(D)**.

Meanwhile, ABGD analysis showed that there was no barcoding gap within Asteropyrum as well and the identified gaps occurred between Asteropyrum and outgroups (Figures 5A,C). The recursive partition of 12 for both markers were obviously unrealistic (Figures 5B,D).

### Niche Differentiation Between Two Morphospecies of Asteropyrum

**FIGURE 6:**
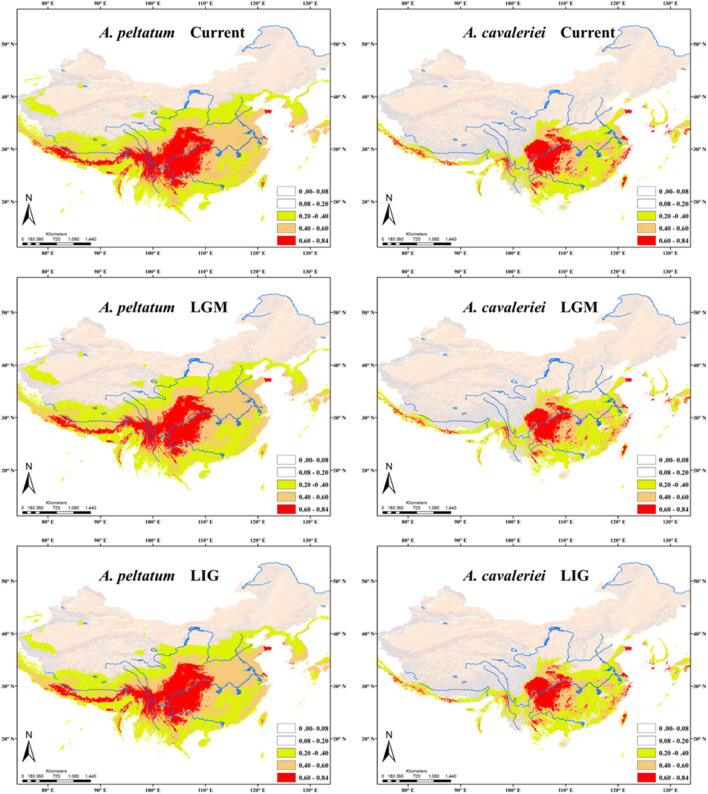
Predicted distributions of *Asteropyrum* using ecological niche modeling. LGM, the Last Glacial Maximum; LIG, the Last Interglaciation.

According to the correlation analysis, fourteen climatic variables (except for bio 4, bio 5, bio 6, bio 10 and bio 15) were retained for niche modeling. Areas under the “Receiver Operating Characteristic (ROC) Curve” (AUC) had values >0.95 for both species, indicating good predictive model performance. The projected distributions of the two morphospecies across different periods changed very little (Figure 6). The Schoener’s *D* values of three climatic periods between these two morphospecies were more than 0.58 (Current 0.614, LGM 0.582, LIG 0.581), reflecting a high overlap of ecological niches. Accordingly, the distribution ranges of two morphospecies were overlapped at different periods (Figure 6), indicating they could have few opportunities to speciate allopatrically. Likewise, principal components analysis (PCA) of 19 bioclimatic variables (Supplementary Table 2) revealed that the two morphospecies of *Asteropyrum* had similar climatic requirements (Figure 3D), although multivariate plots of PC1 and PC2 showed that *A. peltatum* adapts to slightly colder environments than *A. cavaleriei* (Figure 3D) as PC1 was loaded mainly by BIO1 (Annual Mean Temperature, Supplementary Table 2).

## Discussion

### High Variation of Leaf Shape and Size in *Asteropyrum*

We observed high variation of leaf shape and size in *Asteropyrum*, *A. cavaleriei* possessing larger and more obviously lobed leaves than *A. peltatum.* Leaves are photosynthetic organs with many other functions such as transpiration, dissipating heat, and pest defense (Niklas, 1988; Nicotra et al., 2011). Leaf traits, including shape and size, are the results of functional trade-offs that have been resolved in various ways by different species depending on their ecological settings (Nicotra et al., 2011). Such a high number of functional trade-offs is responsible for the tremendous morphological diversity in plant leaves, especially for dicots (Niklas, 1988). Remarkably, different leaf morphology may occur between closely related species, as within-species variants, or even in the same plant (Tsukaya, 2006; Nicotra et al., 2011).

Although high leaf form variation in *Asteropyrum*, we did not find any morphological gap and geographical trend of leave shape (Figures 1B,C). It seems that deeply lobed leaves that traditionally belong to *A. cavaleriei*, generally occur hotter environments at low elevations (below 1,700 m a.b.l.) or southern localities (Figure 1C and Supplementary Table 1). Accordingly, PCA analysis of the 19 bioclimatic variables in *Asteropyrum* indicates that *A. cavaleriei* occurs in environments with higher annual mean temperature (Figure 3D). It has been noted that by adding lobes to a leaf, the rate of heat transfer across a leaf is greater than that of an unlobed leaf of the same area (e.g., Gurevitch and Schuepp, 1990). So, deeply lobed leaves may be selected for hotter environment to reduce the temperature of leaf blades. On the other hand, deeply lobed leaves have a lower ratio of mesophyll tissue to large, highly conductive veins, they have reduced hydraulic resistance relative to less or unlobed leaves (Sack and Holbrook, 2006). Therefore, leaf lobing could represent an effective removal of hydraulic stress-prone tissue and lobed leaves in *Asteropyrum* might be an adaptation to warmer conditions.

Compared to leaf shape that is highly labile and responsive to a range of biotic and abiotic factors, leaf size varies in a more straightforward way: small leaves are associated with harsh conditions such as cold, hot, dry, high light, exposed, nutrient poor and saline environments (Dkhar and Pareek, 2014). In this study, we found that populations with smaller leaves (usually belonging to *A. peltatum*) generally inhabit high elevations or northern localities (e.g., SNJ, PL, Figure 1C), possibly indicative of an adaptation to colder and harsher environments.

In addition to an adaptation to different environments (i.e., ecotypic variation or genetic variation), phenotypic plasticity could also contribute to the variation of leaf shape and size (Royer et al., 2008). We notice that there are both lobed and slightly lobed leaves within the same population such as LCG. Although this variation might be attributed to genotypic difference among individuals, phenotypic plasticity is more likely because we also found such variation in the same individual. In plants, well-developed plasticity of many traits is usually interpreted as an adaptive response to environmental heterogeneity as a consequence of immobility and modular growth (Sultan, 2000). Populations of *Asteropyrum*, predominantly occurring in riparian habitats which are highly heterogeneous in terms of environmental factors such as soil moisture, could have experienced strong balancing selection to maintain polymorphisms over long periods of time within each population (Zhu et al., 2020).

### Integrative Taxonomy of *Asteropyrum* Based on Multiple Lines of Evidence

Plant taxonomists have long recognized the importance of leaf features for identifying taxa. Leaf characters are emphasized because floral features either illustrate little variation or are available only during the relatively short flowering season for each species (Jensen et al., 2002). In fact, for some groups of plants, e.g., *Quercus*, *Betula*, as well as *Asteropyrum* in this case, leaf characters are considered “the most important”. However, as discussed above, the leaf shape and size of *Asteropyrum* are labile and vary among different populations and even within populations. This casts a doubt on the species boundaries of the two species in *Asteropyrum*. Based on a geometric morphometric approach, this study found that the variation of leaf shape and size in *Asteropyrum* is continuous (Figures 1B,C) and these features are not able to discriminate the two species reliably (Figures 3A–C). This conclusion is largely consistent with Yuan and Yang’s (2006) observation. However, our conclusion is more robust because we have investigated the leaf variation across the whole range of *Asteropyrum* and adopted a more powerful tool (i.e., GMM) that can statistically analyze the shape and size variation separately.

Molecular evidence has been increasingly applied in species delimitation because the easy availability and insensitivity to environment changes of DNA sequences. In this study, Automatic Barcode Gap Discovery (ABGD), phylogenetic reconstruction and haplotype network analyses based on plastid *psb*A-*trn*H and nuclear ITS2 sequences all indicate that that there is no barcoding gap within *Asteropyrum* and the haplotypes of both morphospecies are not reciprocally monophyletic (Figures 1, 4, 5). In addition, population genetic assignment based on five low-copy nuclear loci indicates that the two genetic clusters do not correspond to the two morphospecies, enhancing that the two morphospecies in *Asteropyrum* are not real genetic entities. This situation is similarly observed between *Bactrocera invadens* and *Bactrocera dorsalis* (Diptera: Tephritidae) and then the invasive fruit fly *B. invadens* was recommended to be synonymized with *B. dorsalis* (Schutze et al., 2015).

The Schoener’s *D* values and principal components analysis (PCA) of 19 bioclimatic variables (Figure 3D) reveals a high degree of niche overlap between the two *Asteropyrum* morphospecies. The results indicate that the two morphospecies have very similar niches (niche conservatism), implying that lineage separation through niche divergence mediated by disruptive selection (ecological speciation) is unlikely within *Asteropyrum* (Graham et al., 2004). Given the niche conservatism of *Asteropyrum*, speciation within this genus would have fulfilled under a scenario of allopatry caused by a geographic barrier that consists of suboptimal environmental conditions for the species in question (e.g., deserts, mountains, or oceans) (Wiens and Graham, 2005). However, the projected distribution ranges of the two morphospecies at different historical stages (current, LGM, and LIG) exhibit a high degree of overlap, without a geographic gap that can potentially act as a physical barrier (Figure 6). These results suggest that both ongoing allopatric speciation and a secondary contact after historical allopatry within *Asteropyrum* are also unlikely.

It is widely accepted among taxonomists that objectively evaluating several lines of evidence within a formalized framework is the most efficient and theoretically grounded approach for defining robust species hypotheses (De Queiroz, 2007). In this study, we used different approaches (GMM, DNA barcoding, phylogenetic reconstruction, and niche modeling) to evaluate the two-species hypothesis of *Asteropyrum*, the results show that there are no distinct boundaries between the two morphospecies of *Asteropyrum* in terms of leaf shape and size, genetic data and ecological niche. Although two genetic clusters were detected by population genetic analysis, the two clusters mismatch with the two morphospecies. Taken together, the two species *Asteropyrum* defined by leaf morphology do not reflect the divergence pattern within the genus and the present classification should be abandoned. In the future, range-wide population genomic study and in-depth phenotypic investigations would be constructive for delineating the species boundaries and understanding the evolution and speciation within *Asteropyrum.*

## Data Availability Statement

The datasets presented in this study can be found in online repositories. The names of the repository/repositories and accession number(s) can be found in the article/Supplementary Material.

## Author Contributions

ZZ and DF conceived and designed the project. LL, HL, and HW collected the materials. YK and JW were responsible for the lab work. SC and WZ measured the morphological traits and performed the analysis. ZZ and SC wrote the manuscript. All authors read and approved the manuscript.

## Conflict of Interest

The authors declare that the research was conducted in the absence of any commercial or financial relationships that could be construed as a potential conflict of interest.

## Publisher’s Note

All claims expressed in this article are solely those of the authors and do not necessarily represent those of their affiliated organizations, or those of the publisher, the editors and the reviewers. Any product that may be evaluated in this article, or claim that may be made by its manufacturer, is not guaranteed or endorsed by the publisher.

## Footnotes

1
https://bioinfo.mnhn.fr/abi/public/abgd/

2
https://www.fluxus-engineering.com/sharenet.htm

3
www.diva-gis.org

4
http://www.esri.com/software/arcgis/arcgis-for-desktop

## Supplementary Material

Supplementary Figure 1 The morphological characters of leaves, flowers and fruits of *Asteropyrum* **(a–e)**. *A. peltatum* (yellow circle) and *A. cavaleriei* (red circle) occur in the same population of LCG, Rongjing County, Sichuan Province **(a)**.

Supplementary Table 1 Sample information of *Asteropyrum.*

Supplementary Table 2 Variable loadings on the first four principal components.

## References

[B1] Adams D. C. Rohlf F. J. Slice D. E. (2004). Geometric morphometrics: ten years of progress following the ‘revolution’. *Ital. J. Zool.* 71 5–16. 10.1080/11250000409356545

[B2] Cain A. J. (1954). *Animal Species And Their Evolution.* London: Hutchinson University Library.

[B3] Darriba D. Taboada G. L. Doallo R. Posada D. (2012). jModelTest 2: more models, new heuristics and parallel computing. *Nat. Methods* 9 772–772. 10.1038/nmeth.2109 22847109 PMC4594756

[B4] Dayrat B. (2005). Towards integrative taxonomy. *Biol. J. Linn. Soc.* 85 407–415. 10.1111/j.1095-8312.2005.00503.x

[B5] De Queiroz K. (1998). “The general lineage concept of species, species criteria, and the process of speciation,” in *Endless Forms: Species and Speciation*, eds Howard D. J. Berlocher S. H. (New York: Oxford University Press), 57–75.

[B6] De Queiroz K. (2007). Species concepts and species delimitation. *Syst. Biol.* 56 879–886. 10.1080/10635150701701083 18027281

[B7] Dkhar J. Pareek A. (2014). What determines a leaf’s shape?. *Evodevo* 5:47. 10.1186/2041-9139-5-47 25584185 PMC4290414

[B8] Doyle J. Doyle J. L. (1987). A rapid DNA isolation procedure for small quantities of fresh leaf tissue. *Phytochem. Bull.* 19 11–15. 10.1016/j.bse.2009.07.003

[B9] Drummond J. R. Hutchinson J. (1920). A revision of *Isopyrum* (*Ranunculaceae*) and its nearer allies. *Bull. Misc. Inform. Kew* 5 145–169. 10.2307/4107428

[B10] Elith J. Graham C. H. Anderson R. P. Dudík M. Ferrier S. Guisan A. (2006). Novel methods improve prediction of species distributions from occurrence data. *Ecography* 29 129–151. 10.2307/3683475

[B11] Evanno G. Regnaut S. Goudet J. (2005). Detecting the number of clusters of individuals using the software STRUCTURE: a simulation study. *Mol. Ecol.* 14 2611–2620. 10.1111/j.1365-294X.2005.02553.x 15969739

[B12] Excoffier L. Laval G. Schneider S. (2005). Arlequin (version 3.0): an integrated software package for population genetics data analysis. *Evol. Bioinform. Online* 1 47–50. 10.1143/JJAP.34.L418 19325852 PMC2658868

[B13] Fielding A. H. Bell J. F. (1997). A review of methods for the assessment of prediction errors in conservation presence/absence models. *Environ. Conserv.* 24 38–49. 10.1017/S0376892997000088

[B14] Fu D. Z. Robinson O. R. (2001). “Asteropyrum J. R. Drummond & Hutchinson,” in *Flora of China*, vol. 6, eds Wu Z. Y. Raven P. H. (Beijing: Science Press).

[B15] Graham C. H. Ron S. R. Santos J. C. Schneider C. J. Moritz C. (2004). Integrating phylogenetics and environmental niche models to explore speciation mechanisms in dendrobatid frogs. *Evolution* 58 1781–1793. 10.1111/j.0014-3820.2004.tb00461.x 15446430

[B16] Guindon S. Dufayard J. Lefort V. Anisimova M. Hordijk W. Gascuel O. (2010). New algorithms and methods to estimate maximum-likelihood phylogenies: assessing the performance of PhyML 3.0. *Syst. Biol.* 59 307–321. 10.1093/sysbio/syq010 20525638

[B17] Guisan A. Thuiller W. (2005). Predicting species distribution: offering more than simple habitat models. *Ecol. Lett.* 8 993–1009. 10.1111/j.1461-0248.2005.00792.x 34517687

[B18] Gurevitch J. Schuepp P. H. (1990). Boundary layer properties of highly dissected leaves: an investigation using an electrochemical fluid tunnel. *Plant Cell Environ.* 13 783–792. 10.1111/j.1365-3040.1990.tb01094.x

[B19] Hair J. F. Tatham R. L. Anderson R. E. Black W. (1998). *Multivariate Data Analysis, 5th edn.* New Jersey: Prentice Hall.

[B20] Hall T. A. (1999). BioEdit: a user-friendly biological sequence alignment editor and analysis program for Windows 95/98/NT. *Nucleic. Acids Symposium Series* 41 95–98. 10.1021/bk-1999-0734.ch008

[B21] He J. Yao M. Lyu R. D. Lin L. L. Liu H. J. Pei L. Y. (2019). Structural variation of the complete chloroplast genome and plastid phylogenomics of the genus *Asteropyrum* (*Ranunculaceae*). *Sci. Rep.* 9:15285. 10.1038/s41598-019-51601-2 31653891 PMC6814708

[B22] Hebert P. D. N. Cywinska A. Ball S. L. deWaard J. R. (2003). Biological identifications through DNA barcodes. *Proc. Biol. Sci.* 270 313–321. 10.1098/rspb.2002.2218 12614582 PMC1691236

[B23] Hebert P. D. N. Gregory T. R. (2005). The promise of DNA barcoding for taxonomy. *Syst. Biol.* 54 852–859. 10.1080/10635150500354886 16243770

[B24] Holling C. S. (1992). Cross-scale morphology, geometry, and dynamics of ecosystems. *Ecol. Monogr.* 62 447–502. 10.2307/2937313

[B25] Hong D. Y. (2016). Biodiversity pursuits need a scientific and operative species concept. *Biodiv. Sci.* 24 979–999. 10.17520/biods.2016203 34063014

[B26] Hošková K. Pokorná A. Neustupa J. Pokorný P. (2021). Inter- and intraspecific variation in grass phytolith shape and size: a geometric morphometrics perspective. *Ann. Bot.* 127 191–201. 10.1093/aob/mcaa102 32463863 PMC7789106

[B27] Hubisz M. J. Falush D. Stephens M. Pritchard J. K. (2009). Inferring weak population structure with the assistance of sample group information. *Mol. Ecol. Resour.* 9 1322–1332. 10.1111/j.1755-0998.2009.02591.x 21564903 PMC3518025

[B28] Jensen R. J. (2003). The conundrum of morphometrics. *Taxon* 52 663–671. 10.2307/4135538

[B29] Jensen R. J. Ciofani K. M. Miramontes L. C. (2002). Lines, outlines, and landmarks: morphometric analyses of leaves of *Acer rubrum*, *Acer saccharinum* (Aceraceae) and their hybrid. *Taxon* 51 475–492. 10.2307/1555066

[B30] Kimura M. (1980). A simple method for estimating evolutionary rates of base substitutions through comparative studies of nucleotide sequences. *J. Mol. Evol.* 16 111–120. 10.1007/BF01731581 7463489

[B31] Klingenberg C. P. (2011). MorphoJ: an integrated software package for geometric morphometrics. *Mol. Ecol. Resour.* 11 353–357. 10.1111/j.1755-0998.2010.02924.x 21429143

[B32] Kou Y. X. Zhang L. Fan D. M. Cheng S. M. Li D. Z. Hodel G. J. H. (2020). Evolutionary history of a relict conifer, *Pseudotaxus chienii* (Taxaceae), in south-east China during the late Neogene: old lineage, young populations. *Ann. Bot.* 125 105–117. 10.1093/aob/mcz153 31765468 PMC6948213

[B33] Leaché A. D. Koo M. S. Spencer C. L. Papenfuss T. J. Mcguire J. A. (2009). Quantifying ecological, morphological, and genetic variation to delimit species in the coast horned lizard species complex (*Phrynosoma*). *Proc. Natl. Acad. Sci. U. S. A.* 106 12418–12423. 10.1073/pnas.0906380106 19625623 PMC2716385

[B34] Librado P. Rozas J. (2009). DnaSP v5: a software for comprehensive analysis of DNA polymorphism data. *Bioinformatics* 25 1451–1452. 10.1093/bioinformatics/btp187 19346325

[B35] Limsopatham K. Hall M. Zehner R. Zajac B. K. Verhoff M. A. Sontigun N. (2018). A molecular, morphological, and physiological comparison of English and German populations of *Calliphora vicina* (*Diptera: Calliphoridae*). *PLoS One* 13:e0207188. 10.1371/journal.pone.0207188 30507944 PMC6277095

[B36] Loy A. Mariani L. Bertelletti M. Tunesi L. (1998). Visualizing allometry: geometric morphometrics in the study of shape changes in the early stages of the two-banded sea bream, *Diplodus vulgaris* (*Perciformes*, *Sparidae*). *J. Morphol.* 237 137–146. 10.1002/(SICI)1097-4687(199808)237:2<137::AID-JMOR5<3.0.CO;2-Z 29852694

[B37] Lu Z. Sun Y. Li Y. Yang Y. Wang G. Liu J. (2021). Species delimitation and hybridization history of a hazel species complex. *Ann. Bot.* 127 875–886. 10.1093/aob/mcab015 33564860 PMC8225278

[B38] Maddison W. P. (1997). Gene trees in species trees. *Syst. Biol.* 46 523–536. 10.1093/sysbio/46.3.523

[B39] Mitteroecker P. Gunz P. (2009). Advances in geometric morphometrics. *Evol. Biol.* 36 235–247. 10.1007/s11692-009-9055-x

[B40] Nicotra A. B. Leigh A. Boyce C. K. Jones C. S. Niklas K. J. Royer D. L. (2011). The evolution and functional significance of leaf shape in the angiosperms. *Funct. Plant Biol.* 38 535–552. 10.1071/FP11057 32480907

[B41] Niklas K. J. (1988). The Role of phyllotactic pattern as a “developmental constraint” on the interception of light by leaf surfaces. *Evolution* 42 1–16. 10.2307/2409111 28563849

[B42] Peakall R. Smouse P. E. (2012). GenAlEx6.5. *Bioinformatics* 28 2537–2539. 10.1093/bioinformatics/bts460 22820204 PMC3463245

[B43] Peterson A. T. Soberón J. Sánchez-Cordero V. (1999). Conservatism of ecological niches in evolutionary time. *Science* 285 1265–1267. 10.1126/science.285.5431.1265 10455053

[B44] Phillips S. J. Anderson R. P. Schapire R. E. (2006). Maximum entropy modelling of species geographic distributions. *Ecol. Model.* 190 231–259. 10.1016/j.ecolmodel.2005.03.026

[B45] Pritchard J. K. Stephens M. Donnelly P. (2000). Inference of population structure using multilocus genotype data. *Genetics* 155 945–959. 10.1093/genetics/155.2.945 10835412 PMC1461096

[B46] Puillandre N. Lambert A. Brouillet S. Achaz G. (2012). ABGD, Automatic Barcode Gap Discovery for primary species delimitation. *Mol. Ecol.* 21 1864–1877. 10.1111/j.1365-294X.2011.05239.x 21883587

[B47] Rambaut A. (2018). *FigTree v1.4.4.* Available online at: http://tree.bio.ed.ac.uk/software/figtree/ (accessed August 25, 2021).

[B48] Raxworthy C. J. Ingram C. M. Rabibisoa N. Pearson R. G. (2007). Applications of ecological niche modeling for species delimitation: a review and empirical evaluation using Day Geckos (*Phelsuma*) from Madagascar. *Syst. Biol.* 56 907–923. 10.1080/10635150701775111 18066927

[B49] Rohlf F. J. (2010). *Tps Series.* Stony Brook: State University of New York.

[B50] Rohlf F. J. Marcus L. F. (1993). A revolution in morphometrics. *Trends Ecol. Evol.* 8 129–132. 10.1016/0169-5347(93)90024-J 21236128

[B51] Rohlf F. J. Slice D. (1990). Extensions of the procrustes method for the optimal superimposition of landmarks. *Syst. Zool.* 39 40–59. 10.2307/2992207

[B52] Ronquist F. Teslenko M. van der M. P. Ayres D. L. Darling A. Höhna S. (2012). Mrbayes 3.2: efficient bayesian phylogenetic inference and model choice across a large model space. *Syst. Biol.* 61 539–542. 10.1093/sysbio/sys029 22357727 PMC3329765

[B53] Rosenberg N. A. (2004). DISTRUCT: a program for the graphical display of population structure. *Mol. Ecol. Notes* 4 137–138. 10.1046/j.1471-8286.2003.00566.x

[B54] Rossini B. C. Oliveira C. A. M. Melo F. A. G. D. Bertaco V. D. A. Astarloa J. M. D. D. Rosso J. J. (2016). Highlighting *Astyanax* species diversity through DNA barcoding. *PLoS One* 11:e0167203. 10.1371/journal.pone.0167203 27992537 PMC5167228

[B55] Royer D. L. Mcelwain J. C. Wilf A. P. (2008). Sensitivity of leaf size and shape to climate within *Acer Rubrum* and *Quercus Kelloggii*. *New Phytol.* 179 808–817. 10.2307/25150502 18507771

[B56] Sack L. Holbrook N. M. (2006). Leaf hydraulics. *Annu. Rev. Plant Biol.* 57 361–381. 10.1146/annurev.arplant.56.032604.144141 16669766

[B57] Sang T. Crawford D. J. Stuessy T. F. (1997). Chloroplast DNA phylogeny, reticulate evolution, and biogeography of *Paeonia* (*Paeoniaceae*). *Am. J. Bot.* 84 1120–1136. 10.2307/2446155 21708667

[B58] Schoener T. W. (1968). Anolis lizards of Bimini: resource partitioning in a complex fauna. *Ecology* 49 704–726. 10.2307/1935534

[B59] Schutze M. K. Mahmood K. Pavasovic A. Wang B. Newman J. Clarke A. R. (2015). One and the same: integrative taxonomic evidence that *Bactrocera invadens* (*Diptera*: *Tephritidae*) is the same species as the Oriental fruit fly *Bactrocera dorsalis*. *Syst. Entomol.* 40 472–486. 10.1111/syen.12114

[B60] Sites J. W. Marshall J. C. (2003). Delimiting species: a Renaissance issue in systematic biology. *Trends Ecol. Evol.* 18 462–470. 10.1016/S0169-5347(03)00184-8

[B61] Sultan S. E. (2000). Phenotypic plasticity for plant development, function and life history. *Trends Plant Sci.* 5 537–542. 10.1016/S1360-1385(00)01797-0 11120476

[B62] Tamura K. Peterson D. Peterson N. Stecher G. Nei M. Kumar S. (2011). MEGA5: molecular evolutionary genetics analysis using maximum likelihood, evolutionary distance, and maximum parsimony methods. *Mol. Biol. Evol.* 28 2731–2739. 10.1093/molbev/msr121 21546353 PMC3203626

[B63] Tsukaya H. (2006). Mechanism of leaf-shape determination. *Annu. Rev. Plant Biol.* 57 477–496. 10.1146/annurev.arplant.57.032905.105320 16669771

[B64] Viscosi V. Cardini A. (2011). Leaf morphology, taxonomy and geometric morphometrics: a simplified protocol for beginners. *PLoS One* 6:e25630. 10.1371/journal.pone.0025630 21991324 PMC3184990

[B65] Warren D. L. Glor R. E. Turelli M. (2010). ENMtools: a toolbox for comparative studies of environmental niche models. *Ecography* 33 607–611. 10.1111/j.1600-0587.2009.06142.x

[B66] White T. J. Bruns T. Lee S. Taylor J. (1990). “Amplification and direct sequencing of fungal ribosomal RNA genes for phylogenetics,” in *PCR Protocols: A Guide to Methods and Applications*, eds Innis M. A. Gelfand D. H. Sninsky J. J. White T. J. (San Diego: Academic Press).

[B67] Wiens J. J. Graham C. (2005). Niche conservatism: integrating evolution, ecology, and conservation biology. *Annu. Rev. Ecol. Evol. Syst.* 36 519–539. 10.2307/30033815

[B68] Yuan Q. Yang Q. R. (2006). Cytology, palynology, and taxonomy of *Asteropyrum* and four other genera of *Ranunculaceae*. *Bot. J. Linn. Soc.* 152 15–26. 10.1111/j.1095-8339.2006.00546.x

[B69] Zelditch M. L. Swiderski D. L. Sheets H. D. Fink W. L. (2004). *Geometric Morphometrics for Biologists: a Primer.* San Diego: Academic Press.

[B70] Zhai W. Duan X. S. Zhang R. Guo C. Li L. Xu G. X. (2019). Chloroplast genomic data provide new and robust insights into the phylogeny and evolution of the *Ranunculaceae*. *Mol. Phylogenet. Evol.* 135 12–21. 10.1016/j.ympev.2019.02.024 30826488

[B71] Zhu S. Chen J. Zhao J. Comes H. P. Li P. Fu C. (2020). Genomic insights on the contribution of balancing selection and local adaptation to the long-term survival of a widespread living fossil tree, *Cercidiphyllum japonicum*. *New Phytol.* 228 1674–1689. 10.1111/nph.16798 32643803

