# Supplementary material for: Species Delimitation of Asteropyrum (Ranunculaceae) Based on Morphological, Molecular, and Ecological Variation
Source: Front Plant Sci. 2021 Sep 10;12:681864. doi: 10.3389/fpls.2021.681864 (PMC8461316; doi:10.3389/fpls.2021.681864)
Supplement: Supplementary Figure 1 — The morphological characters of leaves, flowers and fruits of Asteropyrum (a–e). A. peltatum (yellow circle) and A. cavaleriei (red circle) occur in the same population of LCG, Rongjing County, Sichuan Province (a). [file Data_Sheet_1.doc]

**Table 1 Sample information of *Asteropyrum***

| Region | | Location | Population Code | Latitude  (ºN) | Longitude  (ºE) | Elevation  (m) | Number |
| --- | --- | --- | --- | --- | --- | --- | --- |
| Burma | Grape | | **PT** | 27.6635 | 97.4266 | 2600 | 1 |
| Tibet | Mêdog | | **MT** | 29.7135 | 95.5821 | 2820 | 12 |
| Yunnan | Fugong | | **FG** | 27.1030 | 98.8190 | 2106 | 12 |
|  | Gongshan | | **GS** | 28.0500 | 98.5667 | 2700 | 12 |
|  | Suijiang | | SJ | 28.3928 | 104.0658 | 1217 | 12 |
|  | Jingdong | | WLS | 24.4156 | 100.7325 | 2200 | 8 |
|  | Wenshan | | WS | 23.3412 | 103.8684 | 2200 | 12 |
|  | Yiliang | | **YL** | 27.8078 | 104.3072 | 1800 | 12 |
|  | Zhenxiong | | **ZX** | 27.3756 | 104.6689 | 2004 | 12 |
| Sichuan | Dujiangyan | | **DJY** | 30.9900 | 103.64 | 2500 | 12 |
|  | Tianquan | | **ELS** | 30.0253 | 102.8603 | 2079 | 12 |
|  | Emei Shan | | **EMS** | 29.5517 | 103.3623 | 2296 | 12 |
|  | Ebian | | HZG | 29.0106 | 103.0561 | 1535 | 12 |
|  | Leibo | | LB | 28.3567 | 103.4825 | 1659 | 12 |
|  | Rongjing | | LCG | 29.6356 | 102.8847 | 1346 | 12 |
|  | Mabian | | MB | 29.0278 | 103.4789 | 1150 | 12 |
| Guizhou | Majiang | | MJ | 26.4183 | 107.335 | 1611 | 12 |
| Shanxi | Pingli | | **PL** | 32.0027 | 109.2798 | 1617 | 12 |
| Chongqing | Youyang | | YY | 29.2533 | 108.9869 | 1290 | 8 |
|  | Wulong | | WL | 29.1958 | 107.3936 | 1577 | 12 |
| Hubei | Shennongjia | | **SNJ** | 31.4434 | 110.3045 | 3057 | 12 |
|  | Xuanen | | XE | 30.0361 | 109.7316 | 1374 | 12 |
| Hunan | Longshan | | LS | 29.5814 | 109.7069 | 918 | 12 |
|  | Sangzhi | | SZ | 29.7674 | 110.0621 | 1310 | 12 |
|  | Xinning | | XN | 26.4064 | 110.9921 | 946 | 12 |
|  | Yongshun | | YS | 28.9936 | 109.8606 | 1310 | 12 |
| Guangxi | Longsheng | | HP | 24.62 | 109.9205 | 846 | 12 |
|  | Ziyuan | | ZY | 25.9117 | 110.414 | 1190 | 12 |
| Total |  | | 28 |  |  |  | 317 |

Bold population codes represent *A. peltatum*, others represent *A. cavaleriei.* Emei Shan indicates Emei Mountains.

**Table** 2 Variable loadings on the first four principal components

| BCV | Explanation | PC 1 | PC 2 | PC 3 | PC 4 |
| --- | --- | --- | --- | --- | --- |
| Bio 1 | Annual Mean Temperature | 0.921 | 0.163 | 0.299 | -0.177 |
| Bio 2 | Mean Diurnal Range | 0.879 | -0.18 | 0.44 | -0.003 |
| Bio 3 | Isothermality | 0.819 | 0.451 | 0.091 | -0.334 |
| Bio 4 | Temperature Seasonality | 0.76 | 0.108 | -0.437 | 0.419 |
| Bio 5 | Max Temperature of Warmest Month | 0.567 | 0.619 | -0.194 | 0.481 |
| Bio 6 | Min Temperature of Coldest Month | 0.642 | -0.605 | -0.419 | 0.077 |
| Bio 7 | Temperature Annual Range | -0.247 | 0.845 | 0.26 | 0.183 |
| Bio 8 | Mean Temperature of Wettest Quarter | 0.617 | 0.573 | -0.263 | 0.466 |
| Bio 9 | Mean Temperature of Driest Quarter | 0.632 | -0.61 | -0.425 | 0.045 |
| Bio 10 | Mean Temperature of Warmest Quarter | 0.349 | 0.795 | -0.078 | 0.452 |
| Bio 11 | Mean Temperature of Coldest Quarter | 0.623 | -0.574 | -0.472 | 0.039 |
| Bio 12 | Annual Precipitation | -0.57 | 0.473 | 0.186 | 0.219 |
| Bio 13 | Precipitation of Wettest Month | -0.444 | 0.763 | -0.159 | -0.123 |
| Bio 14 | Precipitation of Driest Month | 0.16 | -0.791 | 0.433 | 0.391 |
| Bio 15 | Precipitation Seasonality | 0.835 | -0.15 | 0.498 | 0.015 |
| Bio 16 | Precipitation of Wettest Quarter | 0.85 | 0.343 | 0.005 | -0.396 |
| Bio 17 | Precipitation of Driest Quarter | -0.078 | -0.6 | 0.574 | 0.508 |
| Bio 18 | Precipitation of Warmest Quarter | 0.614 | 0.252 | 0.732 | -0.013 |
| Bio 19 | Precipitation of Coldest Quarter | 0.871 | 0.326 | -0.076 | -0.315 |

BCV: Bioclimatic variable

**Figure 1 The morphological characters of leaves, flowers and fruits of *Asteropyrum* (a-e). *A. peltatum* (yellow circle) and *A. cavaleriei* (red circle) occur in the same population of LCG, Rongjing County, Sichuan Province (a).**
